# A Practical Guide to Developing and Troubleshooting Patient-Derived “Mini-Gut” Colorectal Organoids for Clinical Research

**DOI:** 10.3390/mps8050121

**Published:** 2025-10-11

**Authors:** Rex Devasahayam Arokia Balaya, Zahra Heydari, Gobinda Sarkar, Estela Mariel Cruz Garcia, Jose M. de Hoyos-Vega, Eugene Krueger, Lauren Helgeson, Alexander Revzin, Alexandra Ros, Akhilesh Pandey, Lisa Boardman

**Affiliations:** 1 Department of Gastroenterology and Hepatology, Mayo Clinic, Rochester, MN 55905, USA; heydari.zahra@mayo.edu (Z.H.); helgeson.lauren@mayo.edu (L.H.); 2 Department of Laboratory Medicine and Pathology, Mayo Clinic, Rochester, MN 55905, USA; pandey.akhilesh@mayo.edu; 3 School of Medicine, University of Puerto Rico, San Juan, PR 00921, USA; estela.cruz1@upr.edu; 4 Physiology and Biomedical Engineering, Mayo Clinic, Rochester, MN 55905, USA; dehoyos-vega.jose@mayo.edu (J.M.d.H.-V.); Revzin.Alexander@mayo.edu (A.R.); 5 The Biodesign Institute, Arizona State University, Tempe, AZ 85281, USA; alexandra.ros@asu.edu; 6 School of Molecular Sciences, Arizona State University, Tempe, AZ 85287, USA

**Keywords:** patient-derived organoids, colorectal cancer, three-dimensional culture (3D)

## Abstract

Patient-derived organoids (PDOs) have emerged as powerful tools in personalized medicine applicable to both non-malignant conditions and to cancer, where they are increasingly used for personalized drug screening and precision treatment strategies in part due to their ability to replicate tumor heterogeneity. They also serve as study model systems to understand disease mechanisms, pathways, and the impact of ex vivo exposures. We present a detailed step-by-step protocol for generating organoids from normal crypts, polyps, and tumors, including methods for tissue processing, crypt isolation, culture establishment, and the transition from basolateral to apical-out polarity for co-culture and exposure-based studies. The protocol also includes immunofluorescence staining procedures for cellular characterization and quality control measures. Our standardized approach successfully generates organoids from diverse colorectal tissues with high efficiency and reproducibility. This comprehensive guide addresses common technical challenges and provides troubleshooting strategies to improve success rates across different sample types. We believe that this resource will enhance reproducibility in organoid research and expand their utility in translational applications, particularly for personalized medicine approaches in colorectal cancer.

## 1. Introduction

The growing global burden of colorectal cancer (CRC) underscores the urgent need for more predictive and accessible preclinical study models. Although CRC has long been associated with aging populations, recent trends show a worrying rise in early-onset cases. This shift, alongside persistent challenges such as drug resistance and unequal access to personalized treatments, shed light on gaps in current diagnostic and therapeutic strategies. Conventional tumor models including cell lines and mouse models have been instrumental in advancing pre-clinical cancer research [1,2,3]. However, these systems fall short in replicating the cellular complexity, spatial architecture, and microenvironmental dynamics of human tumors [4,5,6].

On the other hand, organoid technology offers a stable and scalable solution that is currently evolving across research laboratories worldwide [7]. Organoids are three-dimensional (3D) cultures that self-organize, are derived from pluripotent stem cells or cancer tissues, and retain the histological and genetic composition of their tissue of origin. Thus, organoids are a viable human model system that incorporates the heterogeneity of tumors in which drug screening and drug dose sensitivity can be performed in an individual with the potential to guide treatment and study mechanisms of resistance in a physiologically relevant context [8,9,10]. With the landmark article by Clevers and colleagues being the first to describe intestinal organoid cultures, they demonstrated that gut stem cells are the most active stem cells in the human body, with the epithelium replacing its cells every 5–7 days [11]. However, researchers have also shown that organoids can be grown from stem cells in other less-proliferative organs [12,13].

The organoid field has been growing continuously, with more than several dozen different colon organoid protocols now described in numerous published studies. The pioneering work by Toshiro Sato and Hans Clevers proved that isolated crypts can form organoid structures recapitulating in vivo small intestinal epithelium, with single Lgr5+ stem cells capable of self-renewal and generating all intestinal epithelial lineages while maintaining the histological hierarchy of native intestinal epithelium in mouse models [14]. Subsequently, Sato, van de Wetering, and Clevers established tumor organoid cultures from 20 consecutive colorectal carcinoma patients, creating a ‘living biobank’ that closely recapitulated the original tumors’ properties, genetic changes, and molecular subtypes [15]. Building upon these foundational studies, other researchers from the Clevers lab developed specialized protocols for molecular biology. Drost et al. employed a CRISPR-based genetic engineering approach in cancer organoids to investigate the origins of mutational signatures [16], and Fujii et al. performed the genetic engineering of human intestinal organoids using electroporation [17], detailing how to maintain self-renewal capacity and cellular diversity in niche-inspired culture conditions [18].

Although numerous protocols for colon organoid generation have been published, a compiled and user-friendly guide for new researchers is still lacking. Existing methodologies are often shaped by specific research goals, spanning applications from developmental biology to translational medicine. As a result, the field has advanced through a diversity of approaches, with protocols varying in cell sources, growth factor combinations, and culture conditions to better replicate the distinct features of colonic epithelial architecture and microenvironmental dynamics. For example Daoud and Múnera used a stepwise differentiation protocol involving Activin A, Wnt3A, FGF4, and CHIR99021 to guide human pluripotent stem cells toward colonic fate, mimicking embryonic development [19]. In contrast, Lee et al. introduced BMP2 activation, along with transcription factors HOXD13 and SATB2, to promote regional identity and maturation in induced pluripotent stem cell (iPSC)-derived colon organoids [20]. Dotti et al. employed adult colonic stem cells embedded in Matrigel and cultured them in a medium supplemented with EGF, Noggin, and R-spondin1 components essential for the long-term expansion and maintenance of epithelial cell diversity [21].

Colon organoid protocols, especially when adapted to generate “apical-out” organoids, provide direct access to the luminal surface, enabling assays of drug permeability, pathogen interactions, barrier function, immune co-cultures that capture epithelial–immune crosstalk, and toxicity research [22,23,24]. Coupling organoids with microfluidics devices helps control the flow, gradient formation, and shear to better mimic the gut milieu and support longitudinal sampling [25,26]. Integration with single-cell sequencing resolves cellular composition and pathway-level responses to drugs [27], while deep-learning based image analysis enables rapid, unbiased phenotyping for patient-specific interpretation [28,29]. Matrix-assisted laser desorption/ionization mass spectrometry imaging (MALDI-MSI) can be used to investigate multiple pharmaceutical compounds in colorectal tumor organoids (CTOs) derived from different patient tissues. This method could be used to predict patient-specific drug responses and help improve the personalized dosing regimens and distributions of lipids and the drug metabolite associated with colon organoids [30,31]. Collectively, these advances enable scalable, high-throughput screening across pharmacologic, microbial, and immunologic applications [32,33,34,35].

## 2. Materials and Methods

### 2.1. Identifying Target Colon Tissue Regions for Sample Collection

Before designing protocols for CRC research, the strategic selection of appropriate sampling sites within the colorectal region is paramount. CRC exhibits significant anatomical heterogeneity, with distinct incidence patterns across different segments of the large intestine. Large-scale epidemiological studies demonstrate that approximately 69% of colorectal cancers occur in the left-sided colon and rectum (distal to the splenic flexure), while 31% arise in the right-sided colon (proximal to the splenic flexure) [36]. Based on anatomical sites of onset, rectal cancer accounts for approximately 50%, and colon cancer accounts for 49% [37]. The study by Zhao et al. reported that among advanced colorectal neoplasms, 34.1% were located in the rectum, 46.8% on the left side, and 19.1% on the right side. A more detailed anatomical breakdown showed that 517 patients (16.6%) had tumors in the ascending colon, 78 (2.5%) in the transverse colon, 36.0% in the descending colon, 10.7% in the sigmoid colon, and 34.1% in the rectum [38]. Furthermore, it has been projected that by 2030, 10% of all colon cancers and 22% of all rectal cancers will be diagnosed in individuals under the age of 50 in the United States [39]. The proximal colon (cecum through transverse colon) demonstrates distinct molecular characteristics, including a higher prevalence of microsatellite instability-high (MSI-H) status, CpG island methylator phenotype-high (CIMP-H), and B-Raf proto-oncogene, serine/threonine kinase (BRAF) mutations, compared with distal colon and rectal cancers [40]. Furthermore, anatomical subsites exhibit distinct etiologies and risk factor profiles, with heterogeneous relationships observed for anthropometric factors, smoking, and other lifestyle variables across proximal colon, distal colon, and rectal locations [41,42]. Understanding this comprehensive anatomical distribution pattern is crucial for guiding the systematic collection of tissue samples—including cancerous, pre-cancerous (polyps), and normal tissues—to ensure representative sampling across the adenoma-carcinoma sequence and enable meaningful organoid-based disease modeling.

**Figure 1:**
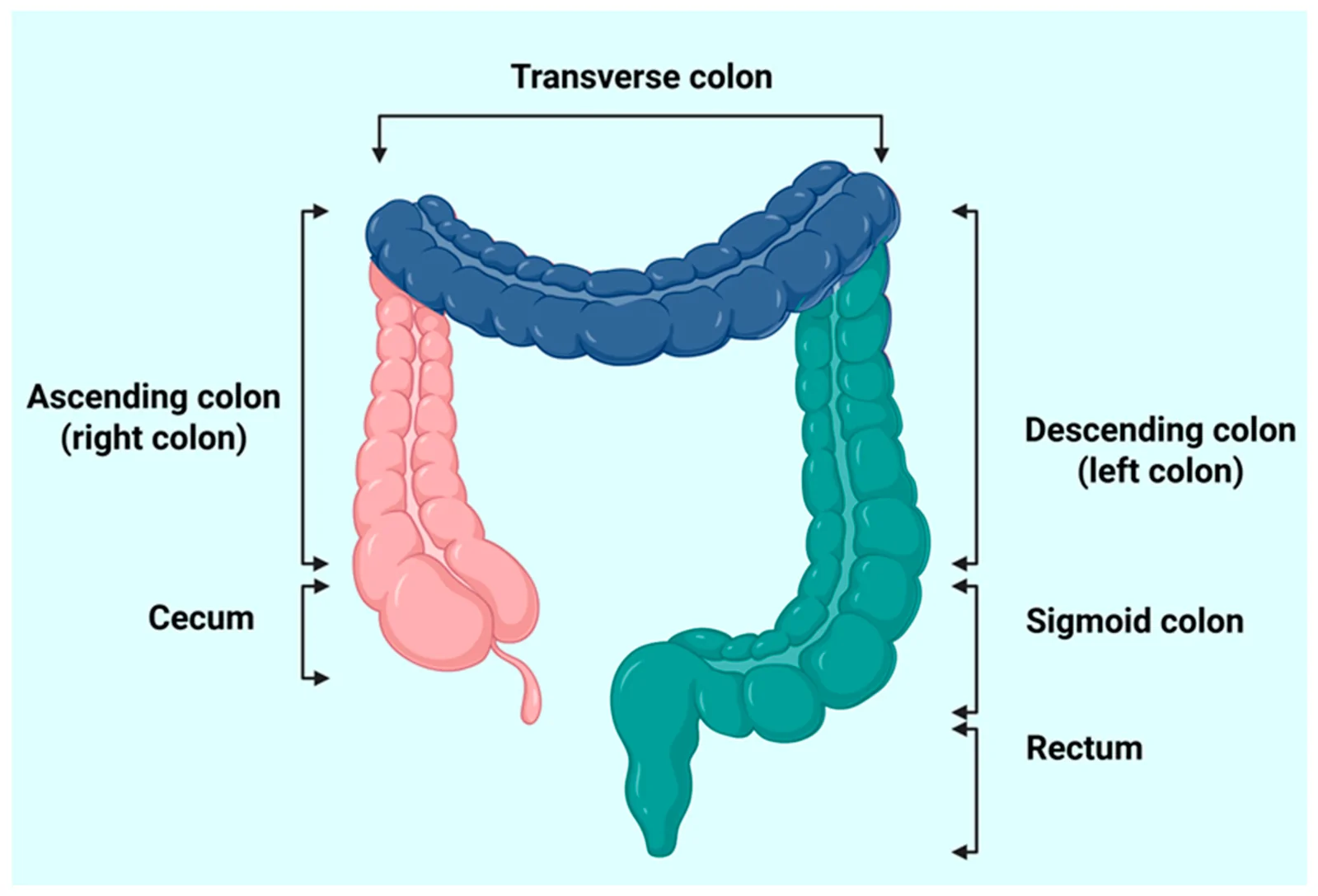
Anatomical sections of the human colon. The large intestine is anatomically divided into distinct segments including the proximal (right-sided) colon, which includes the cecum and ascending colon (shown in pink) as well as the proximal two-thirds of the transverse colon (shown in blue). The distal (left-sided) colon comprises the distal third of the transverse colon, descending colon, and sigmoid colon (shown in green). The rectum represents the terminal of the large intestine (also shown in green). Created with BioRender.com, accessed on 8 August 2025.

This background information enables the appropriate grouping and stratification of samples, especially when analyzing tumor heterogeneity, immune response, or response to drug treatment. It also supports reproducibility and meaningful clinical correlations and facilitates the development of robust biobanking protocols for precision medicine applications in CRC research (Figure 1).

### 2.2. Tissue Procurement and Initial Processing (Approximately 2 h)

Sample collection: Human colorectal tissue samples were collected under sterile conditions immediately following post-procedure (e.g., colonoscopy or surgical resection), in accordance with Institutional Review Board (IRB) approved protocols and after obtaining informed consent from all patients. Prompt handling was performed to ensure the preservation of tissue integrity.


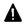
 **CRITICAL STEP:** Transfer samples in a 15 mL Falcon tube containing 5–10 mL of cold Advanced DMEM/F12 medium supplemented with antibiotics (e.g., penicillin-streptomycin) to avoid microbial contamination during transit. Delays in tissue processing reduce cell viability and impact organoid formation efficiency. Cryopreservation is an additional option to preserve the tissues for future organoid development.


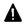
 **CRITICAL STEP:** In some laboratories, same-day sample processing is not possible; this is often a challenge, particularly when there is a disconnect between the clinical site and the research lab. To minimize sample loss and increase reproducibility, we used two methods. (1) Interim cold storage (6–10 h) with antibiotics: if the delay was within 6–10 h, tissue collected at night was given an antibiotic wash, stored at 4 °C in RPMI or DMEM containing antibiotics, and processed the following morning. (2) Cryopreservation: after an antibiotic wash, tissue was cryopreserved in an appropriate medium for later processing. We observed a 20–30% variability in live-cell viability between these two preservation methods. Based on this experience, we recommend selecting the method according to the expected delay; when the delay exceeds 14 h, cryopreserving the tissue and processing it later is preferable. Two validated preservation methods are given below.

Method 1. Short-term refrigerated storage: Wash tissues with antibiotic solution and store at 4 °C in Dulbecco’s modified Eagle medium (DMEM)/F12 medium supplemented with antibiotics.

Method 2. Cryopreservation: Wash tissues with antibiotic solution followed by cryopreservation using an appropriate freezing medium (10% fetal bovine serum (FBS), 10% DMSO in 50% L-WRN (Wnt3a, R-spondin, and Noggin conditioned medium).

Based on our observations, we found 20–30% variability in the cell viability between these preservation methods. Based on our observations, the method selection should be guided by the anticipated processing delay: if ≤6–10 h delay, refrigerated storage at 4 °C is acceptable; tissues may be processed the following morning with minimal viability loss and if there is a ≥14 h delay, cryopreservation is recommended to maintain the optimal cell viability for subsequent organoid culture.

Alternative preservation solution: An additional option for tissue preservation is the use of specialized hypothermic storage solutions such as HypoThermosol FRS (STEMCELL Technologies, Cat No. 50-197-4435), which may provide improved cell viability during extended storage periods compared with standard media-based preservation.

## 3. Cryopreservation

### 3.1. Required Items and Media

- DMEM/F12 (Gibco, Thermo Fisher Scientific; cat. no. 11320033) + 20% fetal bovine serum (FBS) (Gibco, Thermo Fisher Scientific; cat. no. A5670801) + Penicillin/Streptomycin (50 mL conical aliquot) (Gibco, Thermo Fisher Scientific; cat. no. 15140122);
- 1× Phosphate buffered saline (PBS) (Sigma-Aldrich D8537);
- Human colon media: 50% L-WRN conditioned media;
- Sterile filtered FBS;
- Dimethyl sulfoxide (DMSO) (Sigma-Aldrich D2650);
- Normocin (InvivoGen ant-nr-1);
- Gentamicin (Amresco E737);
- Antibiotic antimycotic solution (AB/AM) (GenDEPOT, Cat. No. C980B07);
- Cryogenic vials (Corning Inc. Cat. No. 430488);
- Freezing container (Nalgene Cryo 1 °C Freezing Container, Cat. No. 5100-0001).

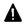
 **CRITICAL STEPS**

Freezing media preparation: 90% FBS + 10% DMSO.

### 3.2. Sample Processing Procedure

1. Transfer samples directly to a 15 mL conical tube containing 5 mL ice-cold PBS. Surgical specimens: Wash with PBS and transfer to a Petri dish on ice.
2. Dissect and remove fat section to isolate the mucosa or cancer tissues, section into small fragments. Transfer processed tissue to a 15 mL conical tube and wash samples twice with chilled PBS and resuspend in 5 mL chilled PBS.
3. Prepare antibiotic solution by adding the following to 5 mL PBS: 10 μL normocin (from 50 mg/mL stock) 5 μL gentamicin (from 50 mg/mL stock), and 50 μL AB/AM (from 100× stock).

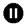
 **PAUSE STEP** Incubate samples in antibiotic solution for 15 min at room temperature.

4. Wash samples twice with chilled PBS, removing all of the supernatant after the final wash.
5. Distribute samples into appropriately labeled cryogenic vials (Lab ID, Tissue Type, Date). Colonoscopy samples: one cryogenic vial per tissue type. Surgical samples: multiple cryogenic vials per tissue type as needed.
6. Add 1000 μL freezing media (given in Step 3) to each sample vial and gently suspend tissue samples in freezing media to ensure uniform distribution. Place cryogenic vials in a freezing container designed to achieve a −1 °C/minute cooling rate. Example: Mr. Frosty™ Freezing Container (Thermo Fisher, Cat no. 5100-0001) or CoolCell™ Freezer Container (Corning^®^, Cat no. CLS432000-1EA). Initial freezing: store container in a −80 °C freezer for 24 h. For long-term storage, transfer vials from −80 °C to liquid nitrogen for long-term cryopreservation.

## 4. Conditioned Medium Preparation

### 4.1. Cell Line and Medium

- The conditioned medium is obtained from a genetically modified L-WRN (ATCC CRL-3276) cell line that simultaneously produces Wnt3a, R-spondin 3, and Noggin, which is extensively utilized in gastrointestinal stem cell cultures.
- L-WRN cells were maintained in high-glucose DMEM supplemented with 10% FBS, 0.5 mg/mL G418 (Geneticin; Cat. No. 10-131-035), and 0.5 mg/mL hygromycin (InvivoGen; Cat. No. ant-hg-1).

**Note:** G418 is unstable in aqueous solutions because it undergoes hydrolysis and degradation, particularly at 37 °C and under repeated freeze–thaw conditions.


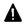
 **CRITICAL STEPS**

Two critical preparatory steps are required prior to the collection of conditioned medium (CM). First, L-WRN cells should be thawed directly into antibiotic-free medium (DMEM supplemented with 10% FBS, without G418 and hygromycin). Second, after 24 h of recovery, the medium should be replaced with complete medium containing G418 and hygromycin to maintain selection pressure.

### 4.2. Step by Step L-WRN Conditioned Medium Preparation Protocol

1. **Cell revival:** Thaw cryopreserved L-WRN cells rapidly at 37 °C. Add directly into pre-warmed DMEM + 10% FBS without antibiotics, and plate immediately in T25 flask. After 24 h, replace with complete medium containing 0.5 mg/mL G418 and 0.5 mg/mL hygromycin B. Optional: Record cell viability at thawing (e.g., Trypan Blue count) to check cell viability.
2. **Passaging:** Subculture when cells are subconfluent (~70–80%) using trypsin-EDTA. Recommended seeding density: 1.5–3 × 10^4^ cells/cm^2^. Change medium every 2–3 days.
3. Maintain L-WRN cells in high-glucose DMEM + 10% FBS, 0.5 mg/mL G418, and 0.5 mg/mL hygromycin B.
4. **Preparation for L-WRN CM collection:** Grow L-WRN cells to confluence in T-150 flasks. Once cells are confluent, switch to collection medium (DMEM + 10% FBS, without G418/hygromycin).
  Note: Rinse the cells with PBS then antibiotic-free DMEM before changing the medium; this will reduce antibiotic carryover.
5. **L-WRN CM collection**
  Allow cells to reach over-confluence (typically it takes 3–4 days). Start collecting CM medium for 4 consecutive days.
  - Remove and discard old medium;
  - Add fresh collection medium;
  - After 24 h, collect medium into a sterile 50 mL Falcon tube.
6. Centrifuge collected medium at 2000× *g* for 5 min at room temperature to pellet cell debris.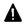 **CRITICAL STEP:** Carefully transfer the supernatant to a new sterile tube (50 mL falcon tube) without disturbing the pellet. Filter the supernatant through a 0.22 µm filter into a sterile container to remove residual debris and contaminants. Label the tube with the date and passage number. Maintain passage records and avoid extended passaging (more than 20 passages can reduce CM activity).
7. Store aliquots at −20 °C for short-term use (within 3 months). For long-term storage, −80 °C is recommended. Avoid repeated freeze-thaw cycles by aliquoting into 50 mL falcon tube.

## 5. Normal Human Colon Crypt Isolation for Organoid Establishment


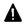
 **CRITICAL STEP**

Preplan: List of things to remember before starting the experiment

- Thaw Matrigel on ice, plan on 45 µL of Matrigel per dome. Thaw time is two to three h for a 1.7 mL Eppendorf aliquot. Alternatively, thaw Matrigel overnight on ice if needed first thing in the morning the next day.
- Note: All of the organoid experiments were performed using Corning Matrigel^®^ Growth Factor Reduced (GFR) Basement Membrane Matrix (Cat. No. 356255). Each lot was accompanied by a certificate of analysis indicating a protein concentration in the range of ~9–12 mg/mL To ensure reproducibility, a minimal lot-acceptance test was performed based on organoid revival efficiency, monitored daily until day 7; only lots that supported robust organoid formation and expansion were retained for downstream experiments.
- Thaw antibiotics on ice.
- Warm a 24-well non-treated plate in a 37 °C incubator.
- Warm human colon media (50% L-WRN CM + 50% Advanced DMEM/F12 with supplements) in a 37 °C water bath for 30 min. (see Table 1 for medium composition).
- Place PBS, DMEM/F12 media, and 10% FBS media on ice.

### 5.1. List of Required Reagents and Materials

- Human colon media: 50% L-WRNCM;
- 1 × PBS (Sigma-Aldrich D8537);
- Normocin (Invivogen ant-nr-1);
- Gentamicin (Amresco E737);
- Antibiotic-antimycotic solution (AB/AM) (Sigma C980B07);
- Collagenase type I (Invitrogen 17100-017);
- Corning^®^ Matrigel^®^ GFR Growth Factor Reduced (GFR) (Product #356231, 1.7 mL aliquots);
- DMEM/F12 without FBS (50 mL conical);
- DMEM/F12 + 10% FBS + Pen/Strep (50 mL conical);
- 24-well non-treated cell culture plate;
- 5 mM EDTA.

### 5.2. Sample Processing Procedure

1. Transfer colonoscopy samples into a 15 mL conical tube containing 5 mL ice-cold PBS. For surgical specimens, rinse with PBS, dissect to isolate the mucosa containing crypts, mince into small fragments, and transfer to a 15 mL conical tube with 5 mL PBS. Add 10 µL normocin, 5 µL gentamicin, and 50 µL AB/AM to 5 mL PBS and incubate tissue in the antibiotic mix for 15 min. Wash samples twice with cold PBS, removing all liquid after the final wash.

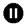
 **PAUSE STEP:** Incubate tissue in 10 mL of 5 mM EDTA at 4 °C on a rocker for 60–75 min. Carefully decant the EDTA and replace it with 5 mL fresh PBS, ensuring that the tissue remains undisturbed. Coat a 10 mL serological pipette with 10% FBS (or FBS/BSA-containing media) to reduce sticking to pipette wall.

**Figure 2:**
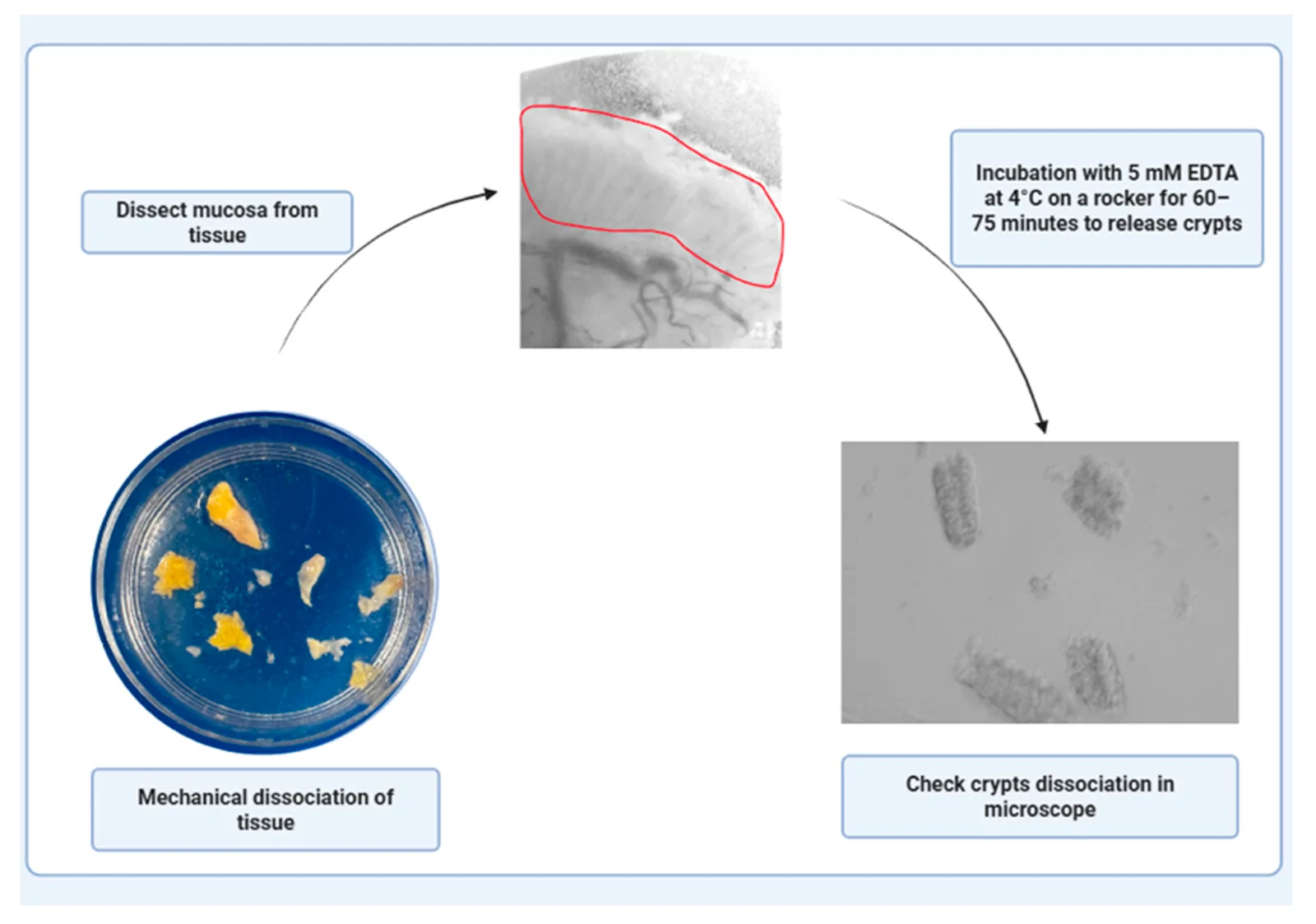
Tissue processing workflow for crypt isolation. The adjacent tissue collected from patients can be used to establish control or normal organoids to be used in the study. First, the dissection of mucosa from tissue samples was performed, followed by incubation with 5 mM EDTA at 4 °C on a rocker for 60–75 min to release crypts from the surrounding tissue matrix. Red circled released crypts were then examined under a microscope to confirm successful dissociation. Created with BioRender.com, accessed on 8 August 2025.

2. Mechanical disruption by pipetting tissue up and down with the coated pipette to release crypts. Check the dissociated crypts under a microscope to confirm successful crypt isolation (Figure 2).

3. Centrifuge at 500× *g* for 5 min at 4 °C.
4. Discard the supernatant carefully, avoiding disturbance of the crypt pellet. Resuspend the crypts in DMEM/F12 (without FBS) using ~10 µL per dome. Pre-wet the pipette tip with DMEM/F12 + 10% FBS before resuspending to reduce cell loss.
5. Mix the resuspended crypts with cold Matrigel (40–50 µL per dome). Plate the cells immediately and avoid introducing bubbles.
6. Pipette 40–50 µL of the Matrigel–crypt mixture into the center of each well in the pre-warmed 24-well plate to form domes. Allow domes to polymerize: 5 min at room temperature and then 20–30 min at 37 °C. Add 750 µL of pre-warmed organoid complete media to each well and incubate.

### 5.3. Key Steps to Remember Before Plating the Crypts

**Figure 3:**
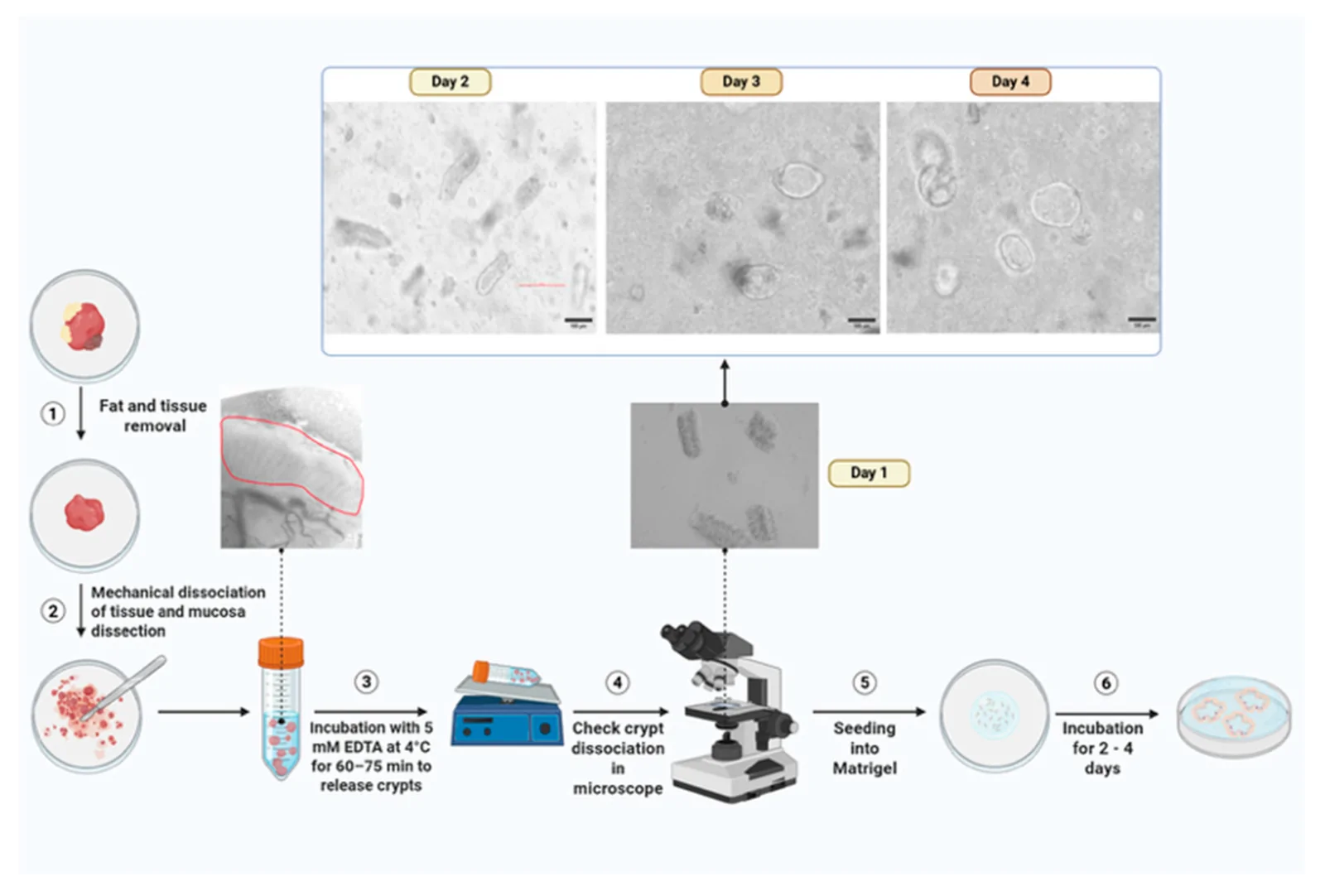
Workflow illustration for establishing patient-derived organoids from cancer-adjusted tissue samples. ① Fat and blood were removed from the tissue sample through careful dissection. ② Mechanical dissociation of tissue was performed to isolate mucosa, followed by ③ incubation with 5 mM EDTA at 4 °C on a rocker for 60–75 min to release crypts from the surrounding tissue matrix. ④ Released crypts were examined under a microscope to confirm successful dissociation. ⑤ Crypts were seeded into the Matrigel matrix in culture wells. ⑥ Organoids were incubated at 37 °C for 2–4 days to allow for establishment and growth. Organoid morphology and development were monitored on days 2–4, showing progressive growth and structural organization. Created with BioRender.com, accessed on 8 August 2025. **Note:** The number of crypts released depends on the thickness and quality of the mucosal layer; we typically use ~100–200 crypts per 40 µL Matrigel dome.

1. Mix pellet with ice-cold Matrigel (approx. 40 µL per dome) carefully to avoid air bubbles.
2. Pipette Matrigel onto the pellet slowly. To avoid introducing air bubbles, do not remove the pipette tip from liquid when pipetting up and down.
3. Pipette 50 µL of the media, Matrigel, and crypts into a well of the pre-warmed plate.
4. Pipette slowly to avoid introducing air bubbles and let the dome polymerize for 5 min at room temperature, then transfer to a 37 °C incubator for 15 min before adding the medium.
5. After polymerization, add 750 µL of 50% L-WRN complete media to each well and place back in the incubator.
6. Check the crypt plate after ~24 h to determine whether the organoids have started to form. Clean and combine wells of crypts to remove tissue debris and make sure the cultures are not too sparse by following the passage protocol to Step 5 and replate.
7. Passage every ~7 days or as needed based on the growth of organoids (Figure 3).

## 6. Polyp/Cancer Cell Isolation

### 6.1. Pre-Experiment Preparation

Follow the same preparation steps as outlined in “Section 5.1. List of Required Reagents and Materials” of the crypt isolation protocol.

**Figure 4:**
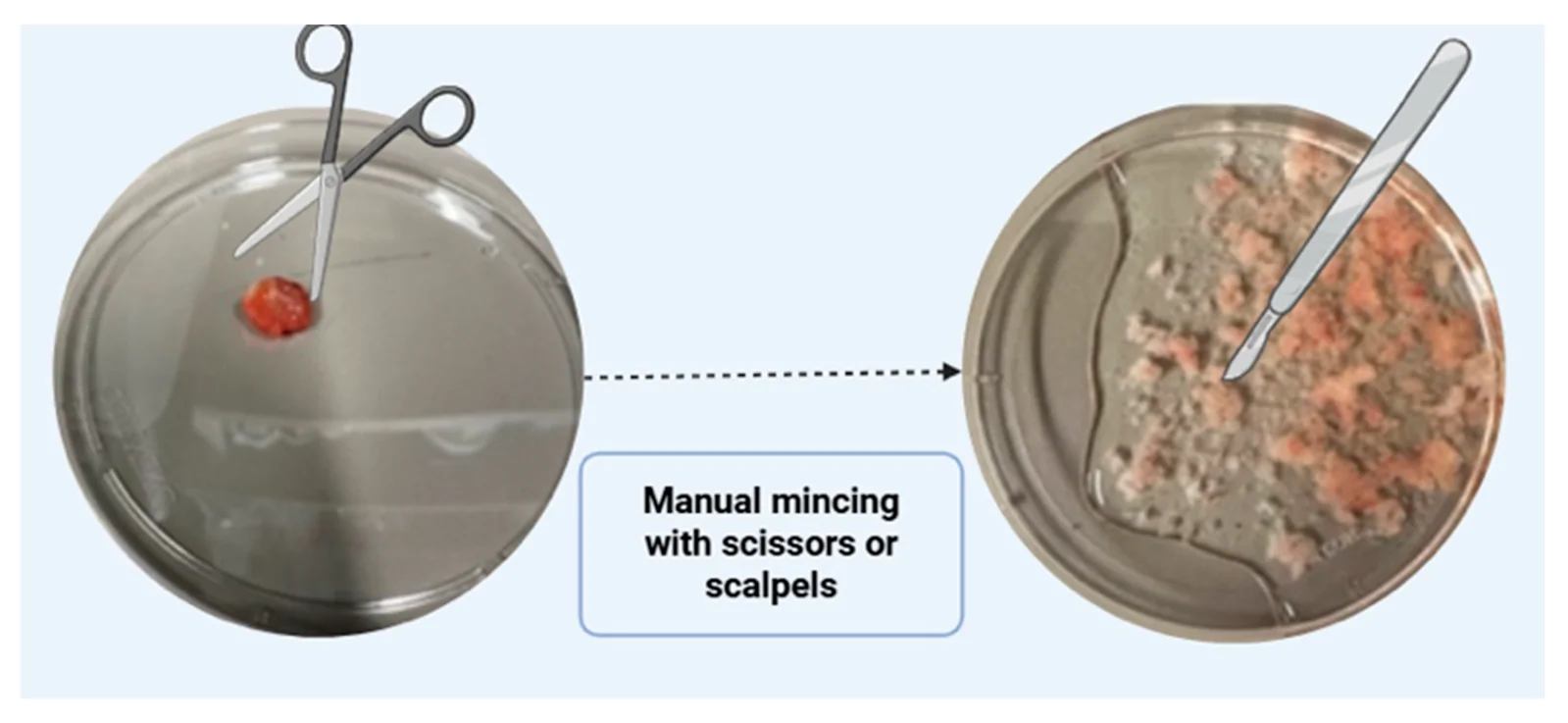
Manual mincing with scissors or scalpels is performed. Mechanical dissociation helps to disrupt the tissues into small fragments, which aids in isolating cells and facilitates organoid formation. Created with BioRender.com, accessed on 8 August 2025.

1. Transfer biopsy samples to 15 mL conical tube with 5 mL ice-cold PBS. For surgical specimens, wash in PBS, dissect mucosa on ice, mince into small pieces, and transfer to a 15 mL tube with 5 mL ice-cold PBS.
2. Incubate sample in antibiotics for 15 min. Add to 5 mL PBS containing 10 µL of normocin (from 50 mg/mL stock), 5 µL of gentamicin (from 50 mg/mL stock), and 50 µL of AB/AM (from 100 × stock). Rinse tissue 2–3 times with cold PBS containing antibiotics to remove blood and debris. Trim away excess adipose tissue using sterile forceps and scissors. Mince cleaned tissue into small fragments using scalpels or fine scissors.

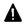
 **CRITICAL STEP:** Avoid over-mincing the tissue. Fragments should be small enough for efficient digestion but not lysed completely (Figure 4).

3. Add 250 μL of collagenase I (6 KU/mL stock; Invitrogen, 17100-017) to 4.75 mL of DMEM/F12 without FBS, yielding a final concentration of ~300 U/mL. For fibrotic or mucus-rich samples, supplement with DNase I (10–20 U/mL) to reduce DNA-mediated clumping. Place the sample in a 37 °C water bath for 20–30 min, agitating gently and monitoring digestion progress under a microscope every 5–10 min. Block the 5 mL collagenase digestion sample by adding 10 mL DMEM/F12 + 10% FBS (1:2 ratio of supernatant to blocking medium).
4. Centrifuge sample at 500× *g* for 5 min at 4 °C. Carefully discard the supernatant without disturbing the cells. Add the desired volume of DMEM/F12 without FBS to the cells.

**OPTIONAL STEP**: Filter the supernatant through a 100 µm filter into a 50 mL Falcon tube.

**Troubleshooting Tip:** Highly fibrotic tumors may require longer digestion or more aggressive pipetting. Monitor digestion under a microscope.

*Note: The volume of media for resuspension depends on the number of domes to be plated. Typically, resuspend in ~10 μL per desired dome, based on a 4:1 Matrigel-to-media ratio. Polyp and cancer samples require high cell density, as they generally form much smaller organoids than normal tissues. For biopsy samples, 2–4 domes per isolation are typical (depending on the number of biopsy “bites” obtained during colonoscopy), while surgical samples typically yield 12+ domes (depending on resection size).


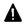
 **CRITICAL STEP:** Pre-wet the pipette tip with DMEM/F12 + 10% FBS and resuspend polyp/cancer cells. Mix pellet with ice-cold Matrigel (approximately 40 μL per dome) carefully to avoid air bubbles because bubbles may interfere with imaging studies.

**Figure 5:**
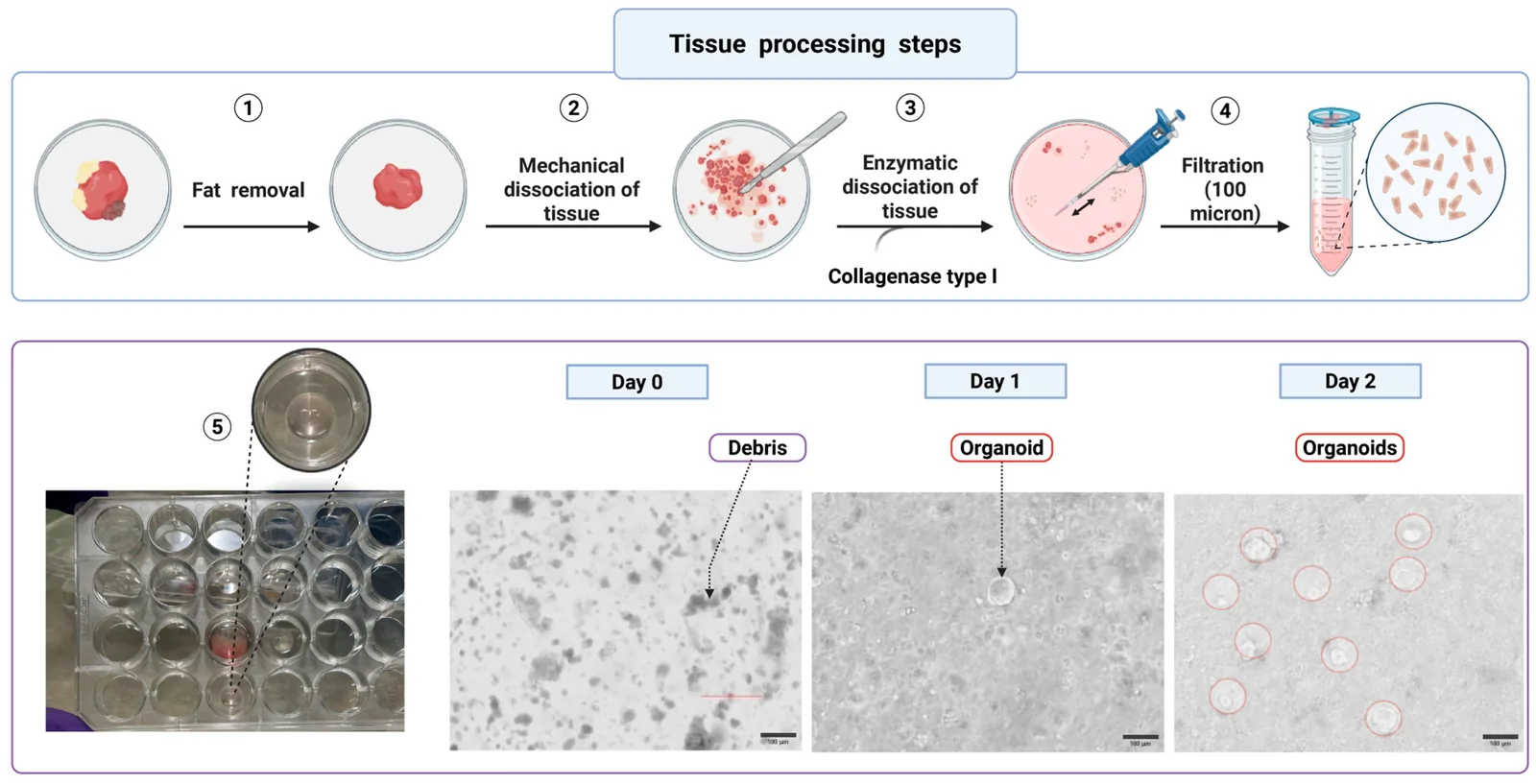
Comprehensive workflow illustration for organoid establishment from cancer tissue samples. ① Fat removal from tissue samples through careful dissection to isolate mucosa. ② Mechanical dissociation of tissue using scissors or scalpels to create small tissue fragments. ③ Enzymatic dissociation of tissue using collagenase type I to further break down extracellular matrix and release individual crypts. ④ Filtration through 100 μm mesh to remove large debris and obtain uniform crypt suspension. ⑤ Seeding of cells in multi-well culture plates containing Matrigel matrix for organoid culture. The bottom panel shows organoid development over time: day 0 (seeding/plating day) through day 2 shows the progression from initial cell seeding to early organoid growth development. Created with BioRender.com, accessed on 8 August 2025.

5. Pipette 50 μL of the media + Matrigel + polyp/cancer cells into a well of the pre-warmed plate.
6. Pipette 40–50 µL of the Matrigel–cells combination into the center of each well in the pre-warmed 24-well plate to form domes. Optional: Invert the well plate to polymerize the dome. Allow domes to polymerize: 5 min at room temperature and then 20–30 min at 37 °C. Add 750 µL of pre-warmed organoid complete media to each well and incubate (Figure 5).

### 6.2. Key Steps to Remember Before Plating the Cells

1. Mix pellet with ice-cold Matrigel (approx. 40 µL per dome) carefully to avoid air bubbles.
2. Pipette Matrigel onto the pellet slowly and do not remove the tip from liquid when pipetting up and down; this will introduce air bubbles.
3. Pipette 50 µL of the media + Matrigel + cells into a well of the pre-warmed plate.
4. Pipette slowly to avoid introducing air bubbles, let the dome polymerize for 5 minutes at room temperature, then transfer to a 37 °C incubator for 15 min before adding medium.
5. After polymerization, add 750 µL of human colon media (50% L-WRN complete media) to each well and place back in the incubator.

## 7. Passage Organoids with TrypLE Express

### Sample Processing Procedure (Typical Passage Once Every 7 Days)

Organoids can be passaged using either mechanical disruption or enzymatic dissociation. Mechanical passaging involves physically breaking up the organoids by pipetting or shearing and is generally faster; however, it often results in variable fragment sizes and reduced reproducibility. In contrast, enzymatic dissociation uses proteolytic enzymes such as TrypLE™ (Cat no. 12605010) to more uniformly degrade the extracellular matrix and separate organoid fragments or single cells.

1. Remove medium from wells (around the solid Matrigel).

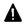
 **CRITICAL STEP:** Direct pipetting will disturb the Matrigel and will lead to organoid loss.

2. Add 500 µL cold Advanced DMEM/F12 (or other base media with no organoid factors) to well and mechanically break up the Matrigel with pipetting P1000 up and down (3–4 times). Repeat this process if needed. The purpose of this step is to wash most of the Matrigel off the wells. Repeat with other wells and combine the domes in a 15 mL Falcon tube.
3. Transfer organoids/media to a 15 mL Falcon tube.
4. Spin down in a refrigerated swing rotor centrifuge at 500× *g* for 5 min at 4 °C.
5. Remove supernatant (note: carefully not to disturb pellet) and resuspend pellet in 500 µL TrypLE Express and put tube in a 37 °C water/bead bath for 10 min. At the 5-minute mark, you can flick the tube (or pipette up and down to break up the organoids if they are particularly mature—this can be checked under a microscope).
  Note: If the sample appears viscous, add DNase I (e.g., 10 U/mL) to aid dissociation.
6. Add 4–5 mL Advanced DMEM/F12 on ice to dilute TrypLE and stop the dissociation of cells and mix the tube.
7. Spin down in a refrigerated swing rotor centrifuge at 500× *g* for 5 min at 4 °C.
8. Remove all supernatants (use 10-mL pipette to remove the supernatant, then the P1000 or P200 pipette, careful not to disturb pellet). Keep the tube on ice.
9. Resuspend the pellet in Matrigel (calculate the amount of Matrigel required, 40–50 µL/well) and plate. After the Matrigel solidifies, add complete 50% L-WRN organoid medium.

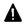
 **CRITICAL STEPS**

**Figure 6:**
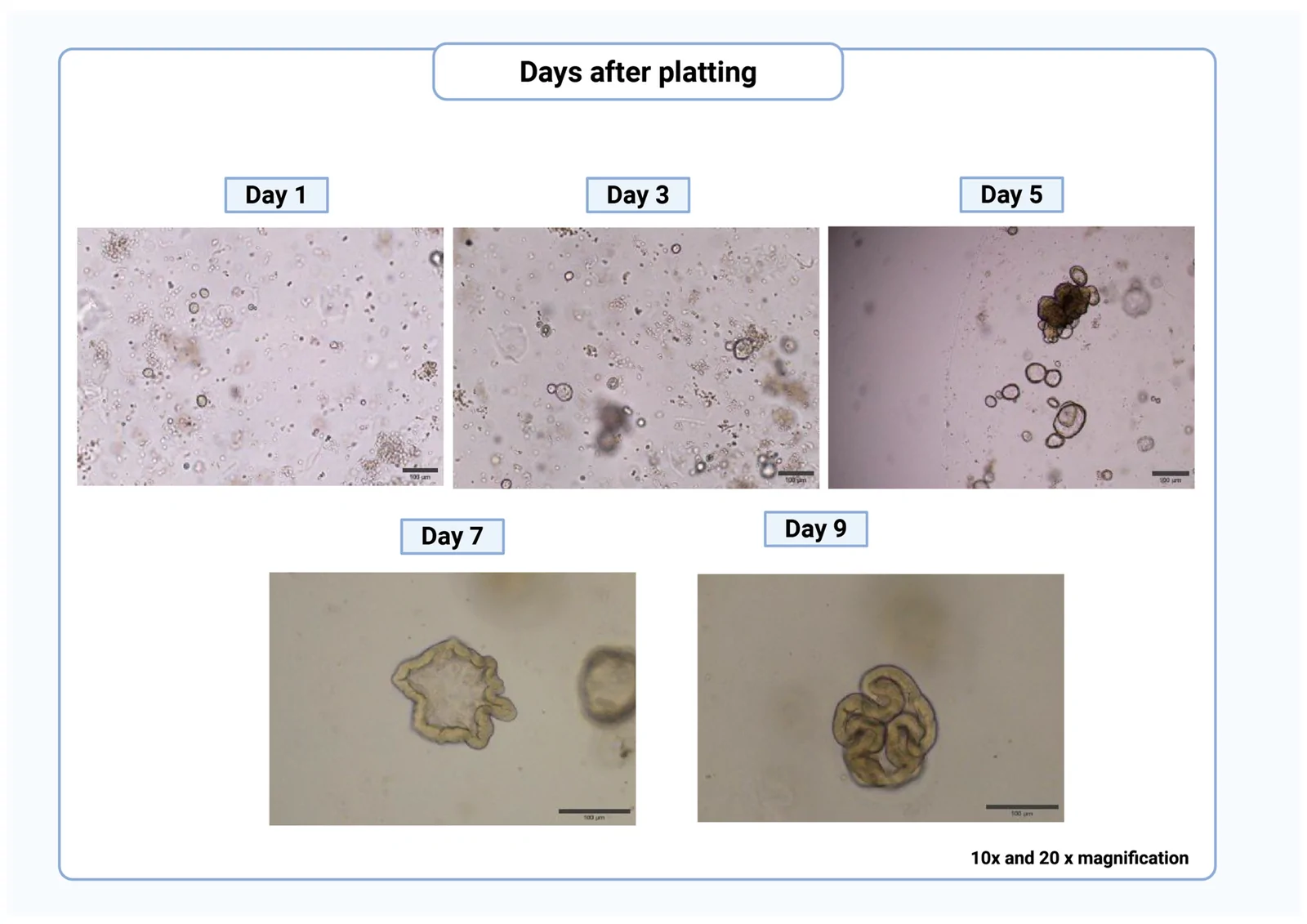
Time-course development of intestinal organoids. Organoid morphology and growth progression are shown at different time points after plating dissociating organoids by using TrypLE express in a Matrigel matrix. Images captured at 10× and 20× magnification demonstrate the progressive development from individual cells. Created with BioRender.com, accessed on 8 August 2025.

Recommended cell split ratio: Seed organoids at a 1:2 to 1:4 ratio, depending on organoid maturity (days 9–11) (Figure 6). and density. The resuspension density is approximately 200–500 cells per 40 µL dome. Add Y-27632 (10 µM) in the culture medium for 24–48 h post-passage to improve cell survival.

## 8. Colon Organoid Culture Medium

The colorectal organoid culture medium consists of Advanced DMEM/F12 supplemented with 50% L-WRN-conditioned medium, antibiotics, and additional growth factors, as detailed in Table 1.

**Table 1:** Reagent for complete colorectal organoid media.

Reagent	Stock Concentration	Volume from Stock (for 50 mL)	Final Concentration	Source	Catalog Number	Role in Colon Organoid Culture
L-WRN conditioned medium (100%)	-	25 mL	50%	In-house	-	Provides Wnt3A, R-spondin, and Noggin signals to maintain stemness and self-renewal and stimulate high levels of Wnt signaling.
Advanced DMEM/F12	-	25 mL	50%	GIBCO (Thermo Fisher Scientific, Waltham, MA, USA)	12634010	Reduced-serum basal medium (1:1 mixture of DMEM and Ham’s F-12) providing essential nutrients for organoid culture.
N2 Supplement	100×	500 μL	1×	GIBCO (Thermo Fisher Scientific, Waltham, MA, USA)	17502048	Basal micronutrients in N2 that support epithelial cell viability and stem/progenitor maintenance in colon PDOs.
B27 Supplement	50×	1000 μL	1×	GIBCO (Thermo Fisher Scientific, Waltham, MA, USA)	17504044	Vitamins and antioxidants enhance cell growth.
EGF	50 μg/mL (1:2)	40 μL	40 ng/mL	R&D Systems (Minneapolis, MN, USA)	236EG200	Activates the EGFR-MAPK signaling pathway to sustain cell proliferation and crypt-like growth of colon PDO epithelium.
SB202190	3 mM	50 μL	3 μM	Sigma-Aldrich (Darmstadt, Germany)	S7067-5MG	p38 MAPK inhibitor, which reduces stress-induced differentiation/apoptosis to maintain colon stem cell compartments.
A83-01	500 μM	50 μL	500 nM	Tocris Bioscience (Minneapolis, MN, USA)	293910	ALK4/5/7 (TGF-β) inhibitor; inhibits the premature differentiation of colon epithelium and supports expansion.
Y-27632	5 mM (5000 μM)	100 μL	10 μM	APExBio (APExBIO Technology LLC, Houston, TX, USA)	501146540	ROCK inhibitor: improves survival of dissociated colon organoid cells after revival or passaging (Note: use for the first 24–48 h post-thaw/passaging).
NAC (N-acetyl-L-cysteine)	0.612 mM (1:1000)	81.5 μL	1 mM	Sigma-Aldrich (Darmstadt, Germany)	A9165-5G	Antioxidant that limits oxidative stress during colon PDO expansion.
Nicotinamide	500 mM	1000 μL	10 mM	Sigma-Aldrich (Darmstadt, Germany)	N3376	Promotes the expansion of colon epithelial progenitors; excessive or prolonged use may dampen differentiation.
Gastrin I	6 μM (1:10)	83.5 μL	10 nM	Sigma-Aldrich (Darmstadt, Germany)	G9020-250UG	Gastrointestinal peptide that supports colon epithelial growth and enhances secretory lineage balance.
Primocin	50 mg/mL	100 μL	100 μg/mL	Invivogen (San Diego, CA, USA)	ant-pm-1	Antimicrobial to prevent bacterial, fungal, and mycoplasma contamination during colon PDO establishment.
Antibiotic/Antimycotic	100×	500 μL	1×	Fisher Scientific (Waltham, MA, USA)	CA002-010	Reduce contamination risk in colon PDO cultures.


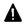
 **CRITICAL STEP:** All media should be sterile-filtered and stored at 4 °C for short-term use (up to 1 week); certain factors such as EGF and A83-01 may degrade and should be added fresh or from aliquots stored at −20 °C. For example, EGF is a small, soluble growth factor that quickly loses biological activity under standard culture conditions. It is susceptible to degradation at 37 °C, and the presence of serum and proteins as well as repeated freezing and thawing cycles further reduce its effectiveness. Adding new EGF to each media change guarantees consistent activation of the EGFR–MAPK pathway, reproducible proliferation, and reduced variability in the colon PDO experiments.

**Note:** Add Y-27632 (10 µM, ROCK inhibitor) during the first 2 days after passaging or thawing to enhance cell survival. Tumor organoids often grow without certain niche factors due to activating mutations (e.g., in *APC*, *KRAS*, *TP53*).

## 9. Apical-Out CRC Organoids

In conventional 3D colon organoids, the apical surface of the epithelium, the functional interface for absorption, secretion, and microbial interactions, is enclosed within the organoid lumen, limiting access for drug exposure or co-culture assays. Although methods such as microinjection or organoid disruption followed by reformation have been used to overcome this, they are technically demanding or may result in variability [43,44].

**Figure 7:**
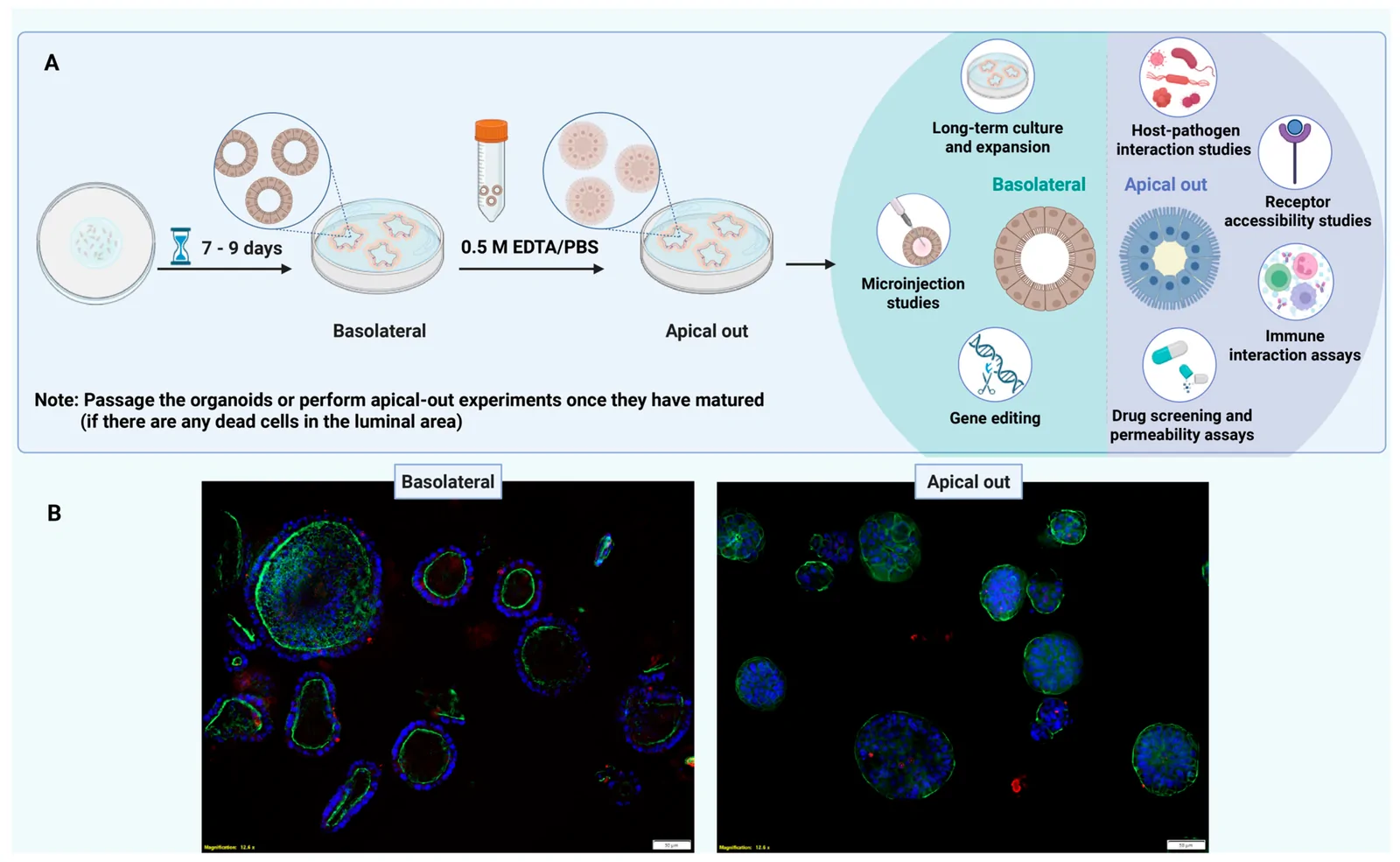
Generation and characterization of apical-in and reverse-polarity (apical-out) colon organoids and imaging using immunofluorescence techniques. (**A**). Diagram illustrating the workflow for generating apical-in and reverse-polarity (apical-out) colon organoids. Cells are isolated from patient-derived samples, enzymatically dissociated, and embedded in Matrigel to form 3D organoids. Organoids were initially cultured within Matrigel domes for 7–9 days, then Matrigel was dislodged from the plate (5 min), followed by incubation with EDTA/PBS for 1 h at 4 °C for matrix removal. Due to the absence of extracellular matrix interactions, the organoids undergo spontaneous eversion, resulting in an apical-out polarity with the apical surface facing outward in the organoid culture medium. (**B**). Representative images showing organoids with basolateral-out polarity embedded in Matrigel (**left** panel) exhibiting internal lumens with apical side facing inward compared with apical-out polarity organoids in suspension (**bottom right** panel), where the apical microvilli face outward. Organoids were counterstained with DAPI (blue) and Alexa Fluor 660 phalloidin (F-actin) (green), both diluted 1:500 for the visualization of nuclei and actin cytoskeleton organization, respectively. Scale bars, 50 µm. This polarity reversal enables direct access to the apical surface for drug testing, infection studies, and functional assays. Created with BioRender.com, accessed on 8 August 2025.

An effective alternative is the generation of apical-out organoids, achieved by releasing organoids from their extracellular matrix and culturing them in suspension medium. Cells are isolated from patient-derived samples, enzymatically dissociated, and embedded in Matrigel to form 3D organoids. Organoids were initially cultured within Matrigel domes for 7–9 days, then Matrigel was dislodged from the plate (5 min), followed by incubation with EDTA/PBS for 1 h at 4 °C for matrix removal; within 2–3 days, the absence of matrix contact induces spontaneous polarity reversal, resulting in organoids with the apical surface facing outward [24]. This results in an apical-out polarity with the apical surface facing outward into the culture medium in the organoid culture medium. This reversal can be confirmed by immunofluorescence staining for apical markers such as ZO-1 or F-actin (stain with phalloidin), indicating correct apical-basal organization (Figure 7). This induces spontaneous eversion, exposing the apical surface outside to the external environment.

### Timeline for Organoid Culture Establishment and Polarity Reversal

Initial tissue processing and culture derivation required 7–9 days from specimen acquisition to the completion of primary passaging (passage 0), with a passaging interval of 7–9 days observed for subsequent culture expansion from passage 0 to passage 1. Following successful culture propagation to passage 2, apical-out polarity reversal was achieved within 3–4 days of protocol initiation. Although morphological reversal was technically possible at passage 2, optimal culture conditions for apical-out organoid derivation were obtained using organoids in the first passage, ensuring sufficient cellular maturation and epithelial integrity prior to polarity manipulation. This standardized protocol yielded the reproducible generation of apical-out organoids within 17–22 days post-specimen acquisition, facilitating consistent experimental design and downstream applications.

## 10. Immunostaining of Organoids

Required Reagents and Solutions

- Fixation solution:
  4% paraformaldehyde (PFA)
  1% Triton X-100 in PBS
  Working solution: 1 mL 16% PFA + 2 mL 2% Triton X-100 + 1 mL PBS
- Washing solution:
  0.1% Triton X-100 in PBS
  Working solution: 1 mL 2% Triton X-100 + 19 mL PBS
- Blocking solution:
  3% BSA + 0.1% Triton X-100 in PBS
- Working solution: 0.3 g BSA + 10 mL PBS + 10 μL Triton X-100
- Dilution buffer (for antibody incubation):
  1× PBS + 1% BSA + 0.1% Triton X-100
  Working solution: 0.1 g BSA + 10 mL PBS + 10 μL Triton X-100

### Step by Step Organoids Processing Procedure

1. Wash organoids (basolateral or apical out) with ice cold PBS.
2. Fix and permeabilize simultaneously at 4 °C through exposure with 4% paraformaldehyde and 1% Triton X-100 in PBS for 3 h.
  - **OPTIONAL STEP**: Remove the supernatant and add 1 × PBS and store at 4 °C if you plan to process the sample the next day. If there is concern regarding epitope masking, perform a 30 min fixation with 4% PFA at room temperature followed by permeabilization with 0.1%Triton to minimize this risk [45,46].
3. Wash solution (0.1% Triton in PBS): Wash the organoids with 0.1% Triton, flush the organoids three times, and then block overnight with 3% BSA and 0.1% Triton in PBS solution at 4 °C.
4. Incubate organoid samples for 24 h at 4 °C with primary antibodies diluted in incubation solution (1 × PBS with 1% BSA and 0.1% Triton X-100).
5. Primary antibody incubation (preparation)
  - Prepare antibodies in dilution:
  600 μL total: 20 μL Phalloidin-iFluor 488 (Abcam, ab176753, Abcam plc, Cambridge, UK) for F-actin, and top up with dilution buffer (PBS + 1% BSA + 0.1% Triton X-100). Incubate organoids for 24 h at 4 °C. Optional: You can also use ZO-1 monoclonal antibody (ZO1-1A12, mouse) instead of Phalloidin-iFluor 488.
6. Wash the samples 5 times and then expose them to secondary antibodies diluted in incubation solution for 24 h at 4 °C.
7. Secondary antibody incubation (preparation)
  - Prepare antibodies in dilution buffer:
8. 80 μL total: 1 μL DAPI solution (1.0 mg/mL, BD Biosciences, 564907, Becton, Dickinson and Company, Franklin Lakes, NJ, USA), and top up with dilution buffer (1 × PBS + 1% BSA + 0.1% Triton X-100). Note: For unlabeled antibodies, use a fluorescent secondary antibody. For example, for ZO-1 (mouse monoclonal), use donkey anti-Mouse IgG (H+L), Alexa Fluor 546 (Thermo Fisher, A10036, Thermo Fisher Scientific, Waltham, MA, USA).Secondary antibody solution was then removed by flushing with washing solutions 5 times.

**Notes:**

- Phalloidin can be added at 1:50 dilution where needed;
- Keep all steps at 4 °C unless otherwise stated;
- Use gentle handling to avoid damaging the organoids;
- Use wide-bore tips to prevent organoid damage;
- Inverted fluorescence microscope settings: Organoids were imaged with an inverted fluorescence microscope (IX-83, Olympus) using 20× long distance objective (LUCPLFLN PH 20 × /0.45). Z stacks were taken in steps of 2 µm and deconvoluted using the constrained iterative process in CellSens Dimensions 3.2 software (Olympus) to remove out-of-focus blur. Images are presented as maximum intensity projection over Z.

**Important note:** Make sure that the Matrigel is completely removed from the organoids and debris, as residual material can cause the cells to clump. Previous studies have reported the use of other basolateral markers such as β-catenin and the apical marker ezrin also used to validate polarity [47,48]. In addition, functional assays such as the 4 kDa FITC-dextran permeability test can be employed [48], in which intact apical-out organoids exclude FITC molecules, whereas barrier disruption results in luminal leakage. Combining immunostaining for polarity markers with functional permeability testing provides a robust approach to confirm epithelial orientation and barrier integrity. This integrated strategy ensures that generated organoids not only exhibit appropriate spatial polarity, but also recapitulate the physiological barrier functions required for host–pathogen interaction studies and therapeutic screening. A common limitation in suspension-based intestinal organoid protocols is the formation of large cell aggregates. These aggregates can compromise experimental efficiency, reduce the reproducibility of organoid morphology, and negatively affect compound permeability and paracrine signaling.

**Note:** We observed that the use of ice-cold medium and keeping organoids on ice facilitated the removal of residual Matrigel, while culturing in ultra-low-attachment plates with gentle orbital shaking reduced the formation of large aggregates.

## 11. Organoid Cryopreservation Protocol

1. Beginning at passage 2, reserve at least two wells from each passage specifically for cryopreservation.
2. Gently scrape the Matrigel domes from the bottom of each selected well using a pipette tip and transfer the contents into a 15 mL conical tube.
3. Rinse each well with 500 µL of complete organoid culture medium to collect any remaining organoids and add the rinse to the same tube.
4. Add additional organoid culture medium to the tube to bring the total volume to 10 mL.
5. Centrifuge the tube at 300× *g* for 5 min at 4 °C.
6. Carefully aspirate the supernatant, leaving a small volume above the Matrigel pellet to avoid disturbing the organoids.
7. Prepare a cryopreservation medium consisting of:
  - 800 µL of complete organoid culture medium;
  - 100 µL of FBS;
  - 100 µL of DMSO (note: use 1 mL of this freezing mixture per two wells).
8. Gently resuspend the organoid pellet in the cryopreservation medium and transfer 1 mL of the suspension into each cryovial.
9. Place the cryovials at −80 °C for a minimum of 24 h. For long-term storage, transfer the vials to liquid nitrogen within a few days.

Note: The freezing medium contains 10% FBS, 10% DMSO, and 80% complete organoid medium (which consists of 50% L-WRN CM + 50% Advanced DMEM/F12). This ensures the presence of Wnt3a, R-spondin, and Noggin during freezing, thereby supporting stem cell maintenance and improving post-thaw recovery of colon organoids.

## 12. Thawing Human Colon Thawing Organoids

### 12.1. Materials Required

- Advanced DMEM/F12;
- 15 mL conical centrifuge tubes;
- Human colon organoid medium: 50% L-WRN-conditioned media;
- Corning^®^ Matrigel^®^ GFR;
- 24-well non-treated cell culture plate.

### 12.2. Sample Processing Procedure

1. Remove the cryovial containing frozen colon organoids from liquid nitrogen storage. Immediately place it in a 37 °C water bath and gently swirl until completely thawed (avoid prolonged exposure).
2. Transfer the thawed contents into a 15 mL conical tube containing 10 mL of room temperature Advanced DMEM/F12 to dilute the cryoprotectant.
3. Centrifuge the tube at 300× *g* for 5 min at room temperature.
4. Carefully aspirate the supernatant without disturbing the pellet.
5. Gently resuspend the organoid pellet in chilled Matrigel. Avoid creating bubbles. Plate the Matrigel drop (dome) into the center of a well in a 24-well plate.

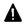
 **CRITICAL STEP:** Typically, the entire thawed vial is plated into one dome, unless it originally contained multiple domes.

6. Leave the plate at room temperature (on the bench) for 5 min to allow the Matrigel to settle. Then, transfer the plate to a 37 °C incubator for 30 min to allow for full polymerization.
7. Once the Matrigel dome has solidified, gently add 750 µL of pre-warmed complete human colon organoid medium to each well.
8. Monitor organoid recovery over the next few days. Change medium if needed. Passage or clean the dome once organoids are healthy and have expanded; timing depends on the specific organoid line. The inclusion of a ROCK inhibitor during the first 24–48 h after thawing to support organoid survival and we advise refreshing the domes once morphological evidence of recovery is observed.

## 13. Recovery and Maintenance During Tissue Processing, Collection Medium Collection, and Co-Culture: Use of Antibiotics

- **Tissue processing:** Antibiotic/antimycotic, normocin, and gentamicin were used to help control bacterial contamination during tissue sample collection and processing.
- **Cells recovery and maintenance (first 24–72 h post-thaw or post-passage):** G418 and hygromycin antibiotics were used only to maintain the L-WRN cells.
- **Washout prior to experiments:** The antibiotics should be removed from culture at least 72 h before initiating conditioned medium (CM) collection or performing microbiome and immune co-culture assays. Cells should be washed twice with PBS, transferred to antibiotic-free medium, and the medium renewed daily for 2–3 days before the experiment.
- **Conditioned medium collection and co-culture assays:** Antibiotics were completely removed during CM production and throughout all of the co-culture experiments. This approach reduces selection pressure, prevents antibiotic carryover into CM, and preserves microbial and immune cell viability and signaling.

## 14. Bridging 3D Organoids to 2D and Organ-on-Chip Platforms

After epithelial expansion, organoids can be used to produce epithelial monolayers for drug testing [49], co-culture experiments [50], and barrier function assessments including transepithelial electrical resistance (TEER) measurements [51,52]. These monolayers can be grown in well plates, Transwells, or microfluidic platforms. Usually, single-cell suspensions are seeded onto collagen type I- or Matrigel-coated substrates to form monolayers, although in vitro planarization of intestinal organoid fragments into epithelium formation has also been reported [50]. The required cell seeding density depends on the cell type and culture platform, generally between 4 × 10^4^ and 2.5 × 10^6^ cells/cm^2^. Fusion of the monolayers can typically be achieved within 48–72 h, and TEER values usually range from 500 to 1500 Ω·cm^2^ [53,54].

Microfluidic culture platforms provide highly organized, controlled, and dynamic microenvironments for epithelial cultures [55,56]. These devices are usually customized to meet specific research needs; however, most share common features such as inlets and outlets, transport microchannels, and culture chambers [57]. Multiple cell types can be co-cultured with epithelial cells in adjacent chambers connected by microchannels or microgrooves, enabling the creation of organ-on-a-chip systems. In addition, gut microbiota interactions with epithelial cells have been demonstrated in several culture platforms including Transwells [58,59] and microfluidic devices [60,61]. The dimensions of these microstructures influence cell–cell interactions including migration, paracrine signaling, and direct intercellular communication through processes such as neurites. Microfluidic cultures can be maintained under static or flow conditions, with the latter requiring external media perfusion systems. Furthermore, microfluidic devices can be integrated with biosensor modules such as TEER electrodes [62]. The epithelial chamber in a microfluidic device can be divided by a porous membrane to establish apical and basolateral compartments, similar to Transwell systems. TEER measurements can be obtained using either external or integrated electrodes, and their values are comparable to, or higher than, those reported in Transwell studies.

## 15. Future Directions

Traditional 2D CRC cell cultures have long been used for drug screening but often fail to mimic the complexity of actual tumors, limiting their predictive value in clinical settings. In contrast, 3D tumor models, particularly patient-derived organoids (PDOs), provide a more accurate representation of tumor biology, making them powerful tools for high-throughput drug screening and personalized therapy development. Studies using PDO biobanks from CRC patients have shown strong correlations between ex vivo drug responses and actual patient outcomes, demonstrating the organoids’ ability to capture tumor heterogeneity. Integrating PDO screening with genomic analyses such as whole exome sequencing (WES) has revealed drug sensitivities linked to specific genetic mutations, paving the way for precision oncology [9]. Immunotherapy has become a central focus of oncological research, catalyzed by several significant advances [63]. The human organoid models represent a physiologically relevant ex vivo system that more accurately recapitulates the tumor microenvironment (TME) and associated immune responses compared with conventional two-dimensional cultures.

In critical cases, organoids allow for a comparison of neoadjuvant and adjuvant immunotherapy, enabling the definition of reaction trajectories, growth dynamics, and emerging mechanistic themes. We have provided a standardized, reproducible, step-by-step protocol for establishing PDOs for colorectal cancer. Workflows include tissue acquisition, crypt isolation, 3D culture, and polarity control, including the generation of apical-out configurations, resulting in in vitro systems that capture the tumor heterogeneity and microenvironmental complexity. These organoid models, particularly those demonstrating apical polarity, provide a solid platform for tumor immunology. They support immune cell co-culture, immune checkpoint inhibitor screening, analysis of tumor–immune interactions, and the functional assessment of TME influence on therapeutic response, ultimately advancing personalized immunotherapy.

Recent advances, including immune cell co-culture, 3D bioprinting, and microfluidic systems, further strengthen TME modeling and facilitate the assessment of immunotherapies such as immune checkpoint inhibitors (ICIs), chimeric antigen receptor (CAR) T-cell therapy, and oncolytic viruses. Despite this promise, challenges remain in representing the diversity of immune cells, maintaining long-term culture stability, and ensuring inter-laboratory reproducibility. Previous PDO colon organoid protocols are well-defined, but beginners often need both a step-by-step workflow and a single, comprehensive guide with troubleshooting and clearly marked critical steps. Our protocol addresses persistent issues in tissue processing, validation, and experimental setups by standardizing pre-analytical variables and helping beginners with a user-friendly catalog-style guide, which can be combined with molecular biology techniques, including CRISPR-based gene editing as well as downstream omics and co-culture studies, to investigate gene function, dissect TME interactions, and advance personalized patient specific therapeutic strategies.

## Data Availability

The original contributions presented in this study are included in the article. Further inquiries can be directed to the corresponding authors.

## Abbreviations

PDOs	Patient-derived organoids
iPSC	Induced pluripotent stem cells
DMSO	Dimethyl sulfoxide
PBS	Phosphate buffered saline
AB/AM	Antibiotic antimycotic solution
DMEM	Dulbecco’s modified Eagle medium
3D	Three-dimensional
MSI-H	Microsatellite instability-high
CIMP-H	CpG island methylator phenotype-high
PFA	Paraformaldehyde
